# Glycosylated Hemoglobin and Albumin-Corrected Fructosamine Are Good Indicators for Glycemic Control in Peritoneal Dialysis Patients

**DOI:** 10.1371/journal.pone.0057762

**Published:** 2013-03-04

**Authors:** Szu-Ying Lee, Yin-Cheng Chen, I-Chieh Tsai, Chung-Jen Yen, Shu-Neng Chueh, Hsueh-Fang Chuang, Hon-Yen Wu, Chih-Kang Chiang, Hui-Teng Cheng, Kuan-Yu Hung, Jenq-Wen Huang

**Affiliations:** 1 Department of Internal Medicine, National Taiwan University Hospital, Yun-Lin Branch, Yunlin County, Taiwan; 2 Department of Internal Medicine, Department of Health, Taipei Hospital, Taiwan; 3 Department of Internal Medicine, National Taiwan University Hospital and College of Medicine, Taipei, Taiwan; 4 Department of Nursing, National Taiwan University Hospital and College of Medicine, Taipei, Taiwan; 5 Department of Nursing, National Taiwan University Hospital, Bei-Hu Branch, Taipei, Taiwan; 6 Department of Internal Medicine, Far Eastern Memorial Hospital, New Taipei City, Taiwan; 7 Department of Integrated Diagnostics and Therapeutics, National Taiwan University Hospital and College of Medicine, Taipei, Taiwan; 8 Department of Internal Medicine, National Taiwan University Hospital, Hsin-Chu Branch, Hsinchu City, Taiwan; University of Sao Paulo Medical School, Brazil

## Abstract

**Purpose:**

Diabetes mellitus (DM) is the most common cause of end-stage renal disease and is an important risk factor for morbidity and mortality after dialysis. However, glycemic control among such patients is difficult to assess. The present study examined glycemic control parameters and observed glucose variation after refilling different kinds of fresh dialysate in peritoneal dialysis (PD) patients.

**Methods:**

A total of 25 DM PD patients were recruited, and continuous glucose monitoring system (CGMS) was applied to measure interstitial fluid (ISF) glucose levels at 5-min intervals for 3 days. Patients filled out diet and PD fluid exchange diaries. The records measured with CGMS were analyzed and correlated with other glycemic control parameters such as fructosamine, albumin-corrected fructosamine (AlbF), glycosylated hemoglobin (HbA1c), and glycated albumin levels.

**Results:**

There were significant correlations between mean ISF glucose and fructosamine (r = 0.45, *P*<0.05), AlbF (r = 0.54, *P*<0.01), and HbA1c (r = 0.51, *P*<0.01). The ISF glucose levels in glucose-containing dialysate increased from approximately 7–8 mg/dL within 1 hour of exchange in contrast to icodextrin dialysate which kept ISF glucose levels unchanged.

**Conclusion:**

HbA1c and AlbF significantly correlated with the mean ISF glucose levels, indicating that they are reliable indices of glycemic control in DM PD patients. Icodextrin dialysate seems to have a favorable glycemic control effect when compared to the other glucose-containing dialysates.

## Introduction

Peritoneal dialysis (PD) is a renal replacement therapy that uses high glucose content to create an osmotic gradient between PD fluid and plasma to achieve ultrafiltration. We previously conducted study to show that higher PD glucose levels had adverse effects on patient survival [1]–[3]. The other effects of glucose absorption via the peritoneum will contribute to hyperglycemia, dyslipidemia, and other metabolic abnormalities in PD patients. However, appropriate glycemic control parameters that can accurately monitor glucose control among PD patients remain to be established. In addition, the real-time glycemic effects of different dialysate are difficult to demonstrate with conventional glucometer or repeated phlebotomy.

Traditionally, glycosylated hemoglobin (HbA1c) has been used to monitor glycemic control in diabetes mellitus (DM) patients. However, HbA1c in chronic kidney disease (CKD) can be influenced by anemia [4] and uremia [5]–[10]. Therefore, HbA1c may not be an ideal measure of blood glucose control in DM CKD patients. Several alternative indices of glycemic control have been reported in literature; these include fructosamine [11], albumin-corrected fructosamine (AlbF) [12], and glycated albumin (GA) levels [13]. All have been reported to accurately reflect glycemic control better than HbA1c levels in CKD patients [14]. However, these studies used only casual fasting blood glucose as the gold standard for comparison with the abovementioned glycemic control parameters.

The continuous glucose-monitoring system (CGMS), which measures interstitial fluid (ISF) glucose levels at 5-min intervals over a couple days, can provide continuous and detailed records of glucose levels of subjects [15]. The CGMS has been validated as a reliable and accurate measurement of blood glucose levels in nonuremic DM patients [16], and it has been recently applied for the assessment of glycemic control in DM hemodialysis (HD) [17] and PD [18] patients.

In this study, we applied the CGMS method to PD patients to test the accuracy of fasting glucose, HbA1c, fructosamine, AlbF, and GA as glycemic control parameters. We found that both HbA1c and AlbF were good indicators of glycemic control in PD patients. In addition, the glycemic effects of variable dialysate were also demonstrated by CGMS.

## Materials and Methods

### Patient Data Collection

From June 2010 to October 2011, DM patients aged more than 20 years who underwent maintenance PD for more than 3 months were enrolled in our study. We excluded patients who had undergone a prior renal transplant, and those who had been newly identified DM after PD. We also excluded patients who could not operate a capillary glucometer. All these patients fitted the DM diagnostic criteria of the American Diabetes Association [19]. In brief, patients with an HbA1c ≥6.5%, fasting plasma glucose level ≥126 mg/dL, or 2-h plasma glucose ≥200 mg/dL during an oral glucose-tolerance test at diagnosis and who had received stable anti-diabetic treatment for minimum 6 months were diagnosed with diabetes. Information collected from these participants included demographic data, and data on height, weight, PD modality, glucose concentration of dialysate, and the medication for DM. Peritoneal membrane transport characteristics were defined based on the results of the most recent peritoneal equilibration test (PET) [20]. Residual renal function was measured by calculating the renal Kt/V with a 24-h urine collection. PD adequacy was measured as the sum of peritoneal Kt/V and renal Kt/V. A fasting blood sample was obtained before implanting the CGMS device whichever dialysate in the peritoneal cavity. The blood was centrifuged immediately at 4°C with 3000 rpm. It was then stored at –80°C until measurement.

We collected the information of the monthly doses of erythrocyte stimulating agents (ESA) three months before CGMS. Potency of darbepoetin alfa (NESP, Taiyo Pharmaceutical Industry, Japan) and methoxy polyethylene glycol- epoetin beta (Mircera, Roche Diagnostics GmbH, Germany) were converted to international unit (IU) with a ratio of 1 µg = 200 IU as epoetin beta (Recormon, Roche Diagnostics GmbH, Germany) [21], [22].

### Ethical Considerations

This study was approved by the ethic committees of National Taiwan University Hospital and Taipei Hospital, Department of Health. The approval numbers were NTUH-REC No. 200912044R and THIRB-10-08, respectively. Patients provided written informed consent before participating in the study.

### CGMS Data Collection

Over the past decade, several CGMS have been developed [23], and these equipments measure variations in the glucose levels continuously over a couple days as a Holter system [15]. This demonstrates the true pattern of glucose levels other than the spot levels of fasting or postprandial glycemic levels. Analysis of the CGMS data provides more information about the efficacy of anti-diabetic agents or insulin regimen [24].

After adequate informed consent, enrolled patients were implanted with a “Medtronic MiniMed” CGMS (Medtronic MiniMed, CA). Through a needle-type glucose sensor inserted subcutaneously into the abdominal wall, CGMS could be used to record ISF glucose levels, which represent the blood glucose levels. The monitor recorded ISF levels every 10 s and then stored a smoothed average over 5 min. The range of ISF detection was 40–400 mg/dL. Glucose oxidase reaction was applied to measure glucose concentration; this serves as a suitable monitor for patients using icodextrin solution [15].

During CGMS implantation, patients were asked to measure capillary glucose 4 times a day and record the time and content of any oral intake. The time of PD fluid exchange and glucose concentration of dialysate were also recorded. After 3 days of continuous measurement, patients returned to the hospital to remove the device; at that time, the results were downloaded and patients submitted their diet and PD fluid exchange diaries.

Mean glucose level within the first hour of exchanging dialysate (Glu _0–1 h_) was calculated by measuring the area under the curve (AUC) in this period. Data from patients who had food intake within 1 hr before or after refilling PD fluid were excluded to avoid any interference of diet. Three-day AUC of ISF glucose levels was also calculated to represent the mean 3-day glycemic levels and compare with other glycemic control parameters.

### Glycemic Control Parameters

Samples drawn preceding CGMS insertion were checked for levels of the following: fasting glucose, HbA1c, insulin, fructosamine, and GA. HbA1c was analyzed on a cation exchange column chromatograph using an automated high-pressure liquid chromatography instrument (HLC-723 G7, Tosoh Corporation, Tokyo, Japan). This HbA1c assay would be elevated to falsely high by carbamylated hemoglobin [25]. Commercial kits of Cobas Mira (Roche Diagnostics, Mannheim, Germany) were used to determinate fructosamine level which had the ability to reduce nitroblue tetrazolium in alkaline medium. The rate of formation of formazane was directly proportional to the fructosamine concentration and was measured photometrically at 552 nm. We determined GA by commercial kits (Exocell Inc. Philadelphia, PA) with monoclonal antibodies that specifically recognized the glycated lysine residue in GA which was also ketoamine formed by a non-enzymatic oxidation of glucose. These measurements were conducted according to manufacturers’ protocols. The method of correction of fructosamine by albumin was described as following:
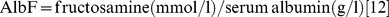



### Dialysate Effects on Glycemia in PD

**Figure 1:**
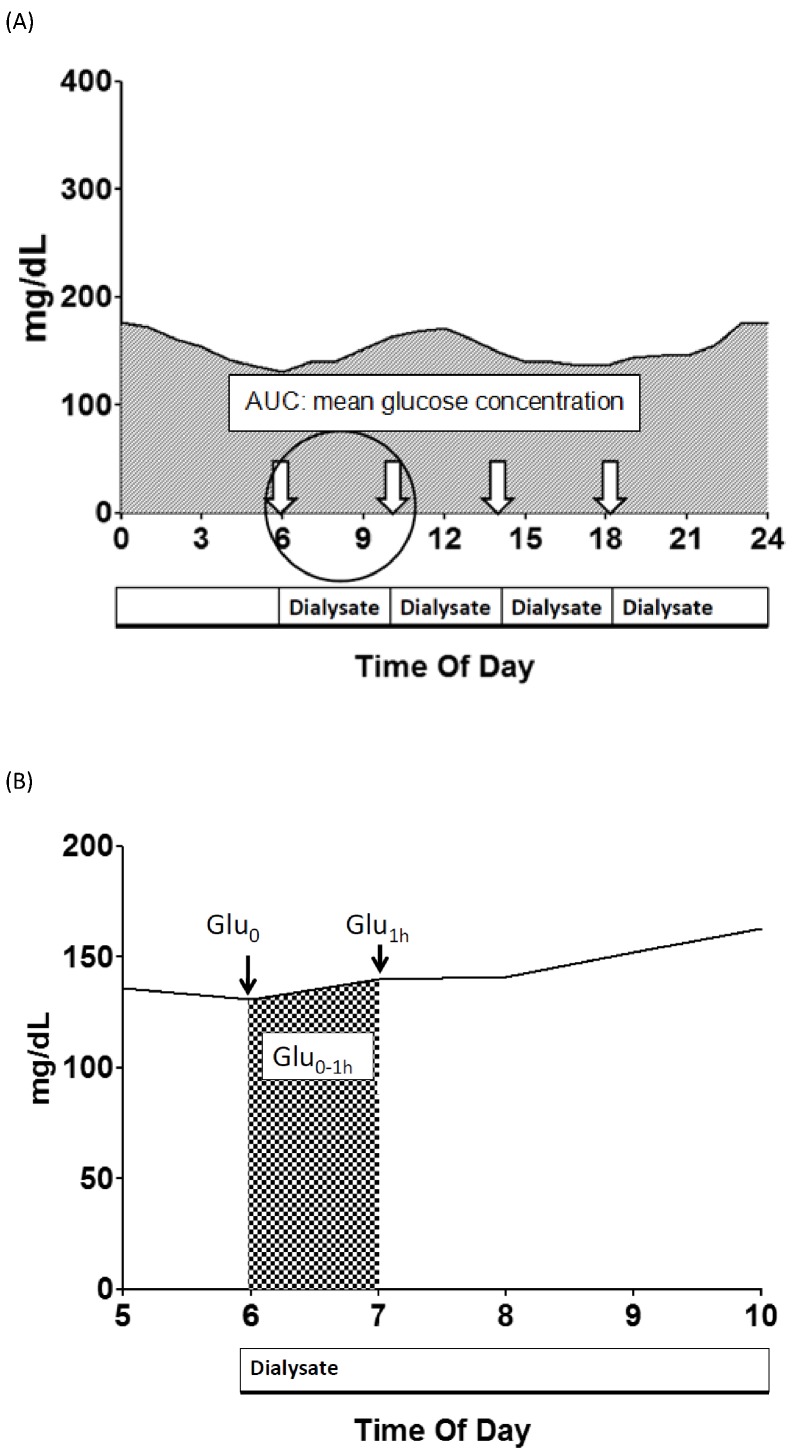
The symbols used to represent the glucose level change by continuous glucose monitoring system.

Different concentrations of glucose-based dialysate including 1.36%, 2.25% and 3.86% were available for ultrafiltration in PD therapy. Thus, we analyzed the short-term glycemic variations among different PD solutions. We defined 4 parameters. First, baseline glucose levels (Glu_0_) was used to represent the glucose level at the time of refilling fresh dialysate, and this signified a baseline glucose level. Second, Glu_1 h_ – Glu_0_, difference between the glucose level at 1 hr (Glu_1 h_) after PD fluid exchange and base line Glu_0_, represented the magnitude of change in ISF glucose concentration within the first hour. Third, Glu_0–1 h_ calculated from the AUC within the first hour after refilling fresh dialysate represented the average ISF glucose concentration in this period as the above mentioned. Fourth, Glu_0–1 h_ – Glu_0,_ difference between mean ISF glucose in first hour Glu_0–1 h_ and the baseline Glu_0_ level, represented the mean glycemic effects of PD fluid within the first hour (Figure 1).

### Statistical Analysis

All variables are reported as mean ± SD or median (25%, 75%) where appropriate for continuous variables and as frequencies or percentages for categorical variables. Student’s *t*-test or non-parametric *t*-test was used for analysis between groups, wherever appropriate. Differences in frequency were tested using Chi-square analysis. Relationships between variables were tested with Pearson correlation. *P* values <0.05 were considered significant. All statistical analyses were conducted using SPSS 13.0 for Windows (SPSS Inc., IL, USA).

## Results

### Clinical Characteristics and Glycemic Parameters

A total of 25 DM patients were recruited in this study. The demographic data and clinical characteristics of these patients are shown in Table 1. Forty-eight percent of study participants were women, and the mean age of the participants was 59±13 years. They had body mass index of 24.7±3.4 kg/m^2^ with mean PD vintage of 18±14 months. Their glycemic control parameters were shown in Table 2. Mean values for serum fasting glucose, HbA1c, fructosamine, AlbF, and %GA were 187±82 mg/dL, 8.1±1.4%, 368±64 µmol/L, 972±203 µ mol/g, and 1.72±1.56%, respectively. The mean ISF glucose level calculated from 3-day AUC of glucose levels measured by CGMS was 215±53 mg/dL. Our patients were relatively more obese, and their glycemic controls were poor. The mean doses of ESA at the month of CGMS and one, two and three months prior to CGMS were 14880±6508 IU, 12320±8035 IU, 11840±6780 IU, and 15920±6041 IU, respectively. There was no difference among these groups (*P* = 0.138 with non-parametric t-test). The doses of ESA had not changed before the preceding 3 months of the study.

**Table 1:** Demographic data and clinical characteristics of the enrolled diabetic peritoneal dialysis patients.

	Mean±SD
Sex (man/woman)	13/12
Age	59±13
Body mass index (Kg/m^2^)	24.7±3.4
Dialysis modality (CAPD/APD)	16/9
PD vintage (months)	18±14
D4/D0 glucose	0.40±0.07
4 hr D/P creatinine	0.67±0.10
Peritoneal Kt/V	1.78±0.36
Renal Kt/V	0.17±0.22
Total Kt/V	1.95±0.38
nPCR (gm/Kg/day)	0.88±0.19
UN(mg/dL)	55.3±15.2
Creatinine (mg/dL)	10.8±3.2
Albumin (gm/dL)	3.8±0.8
Hb (g/dL)	10.2±1.8
Cholesterol (mg/dL)	212±55
Triglyceride (mg/dL)	157±194
LDL(mg/dL)	99±39
HDL(mg/dL)	44±10
CRP(mg/dL)	0.77±0.87

Continuous ambulatory peritoneal dialysis (CAPD).

Automated peritoneal dialysis (APD).

Normalized protein catabolic rate (nPCR).

**Table 2:** Glycemic control parameters among the recruited peritoneal dialysis patients.

	Mean±SD
Fasting glucose (mg/dL)	187±82
HbA1c (%)	8.1±1.4
Fructosamine (umol/L)	368±64
Albumin-corrected fructosamine(umol/g)	972±203
Glycated albumin %	1.72±1.56
3-day mean glucose AUC by CGMS (mg/dL)	215±53
Insulin (µU/mL)	13.95±6.23

AUC: area under curve.

Continuous glucose monitoring system (CGMS).

Diet/antidiabetic agent (ADA)/ADA+insulin/insulin (n): 3/4/6/12.

### Glycemic Control Parameters Correlate the Glucose Levels Measured by CGMS

**Figure 2:**
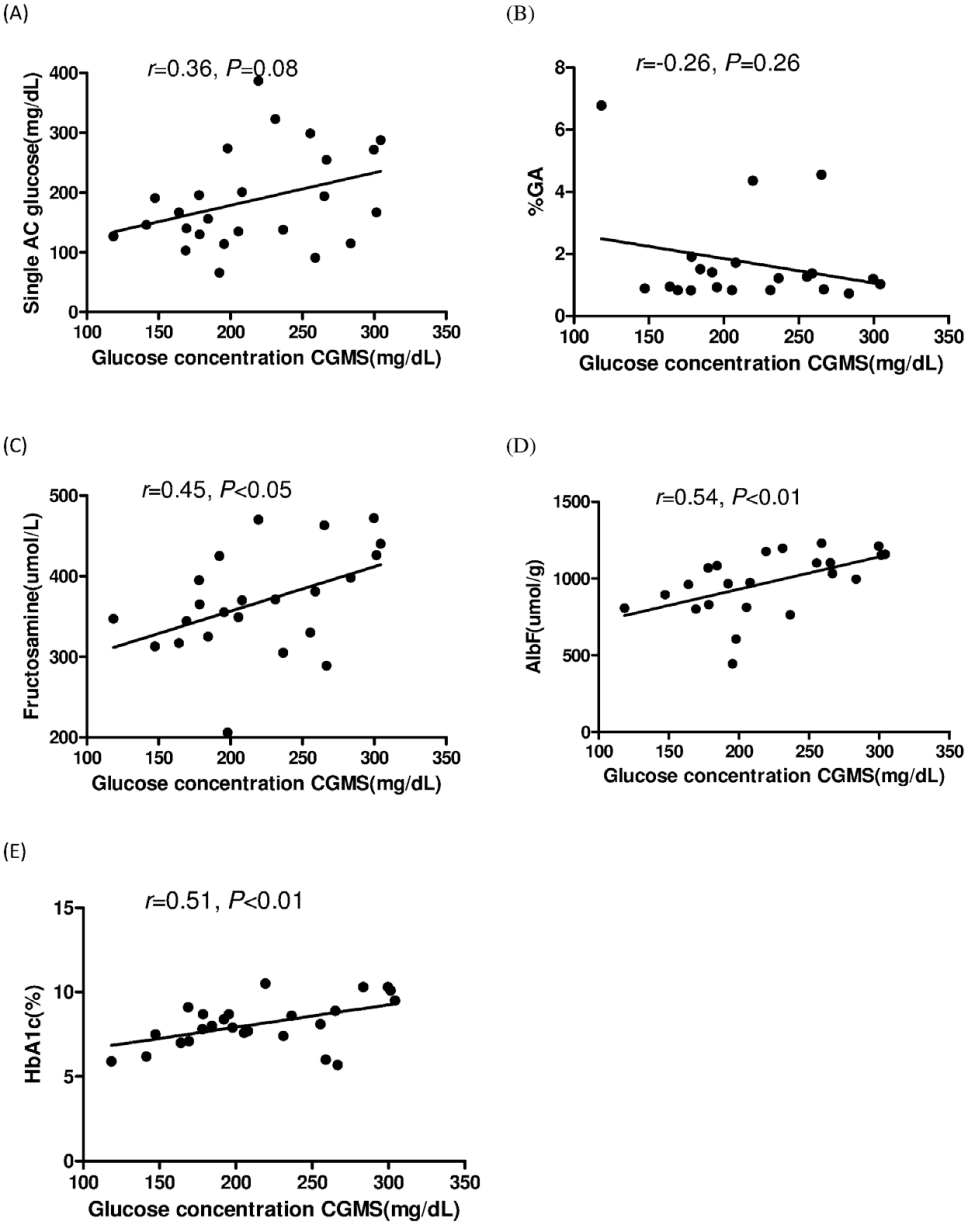
Correlation between ISF glucose and glycemic control parameters. Correlation between 3-day mean interstitial fluid glucose levels measured with continuous glucose monitoring system and levels of single-fasting serum glucose (A), glycated albumin percent (B), fructosamine (C), albumin-corrected fructosamine (D), and glycosylated hemoglobin (E).

To test whether glycemic control parameters could predict chronic glucose control in PD patients, we analyzed the relationships between the data measured by CGMS and other clinically used glycemic control parameters (Figure 2). 3-day mean AUC of glucose levels were significantly correlated with fructosamine (r = 0.45, *P*<0.05), AlbF (r = 0.54, *P*<0.01), and HbA1c (r = 0.51, *P*<0.01). However, there was no correlation between mean AUC and single serum fasting glucose (r = 0.36, *P* = 0.08) or %GA (r = –0.26, *P* = 0.26). These results suggested that HbA1c and AlbF could represent chronic glucose control accurately in PD patients.

### Glycemic Change within the First Hour of Dialysate Exchange

**Figure 3:**
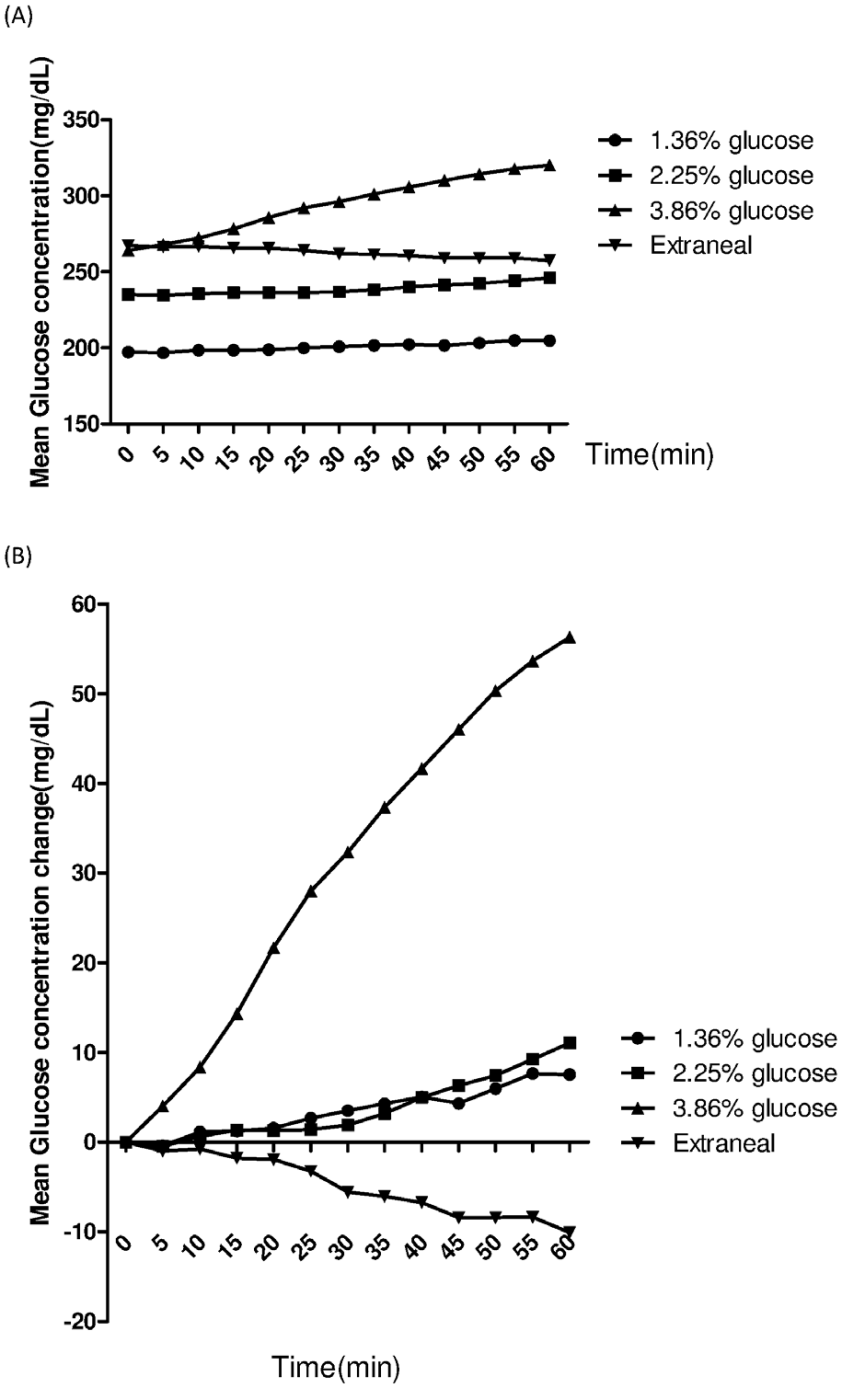
Glucemic change within the first hour of dialysate exchange. The time course of interstitial fluid (ISF) glucose levels (A), and the change of ISF glucose levels (B) within the first hour of refilling fresh dialysate among different kinds of dialysates, including 1.36%, 2.25%, 3.86% glucose dialysate, and Extraneal.

Next, we assessed short-term change in glucose levels during PD fluid exchange. There were 16 continuous ambulatory peritoneal dialysis (CAPD) patients enrolled in our study. ISF glucose variations within 1 h of refilling fresh dialysate were further analyzed, and these variations were shown in Fig. 3A. Since the Glu_0_ for each dialysate were not identical, the changes in ISF glucose concentrations within the first hour (Glu_1 h_ – Glu_ 0_) of exchanging fresh dialysate were studied (Fig. 3B). The levels of change were similar between the 1.36% and 2.25% glucose dialysate. However, there were prominent increments in ISF glucose levels after exchanging 3.86% glucose dialysate. Icodextrin dialysate administration had no effect or even lowered the ISF glucose levels.

There was a trend towards increased ISF glucose concentration as the dialysate glucose increased (Table 3). Since CGMS had serial glucose measurement, mean ISF glucose level within 1 h (Glu_0–1 h_) and the mean increment of glucose levels within 1 h (Glu_0–1 h_ – Glu_0_) could be calculated with these data. Glu_0–1 h_ was highest after 3.86% glucose indwelling, but the sample number was only 3. Glu_0–1 h_ – Glu_0_ was similar for 1.36% and 2.25% glucose dialysate use, and icodextrin had the lowest increment of ISF glucose levels (Table 3).

**Table 3:** The change in interstitial fluid glucose concentration within the first hour of peritoneal dialysis fluid exchange among different kinds of dialysates.

	1.36% glucose	2.25% glucose	3.86% glucose	Extraneal
n	53	77	3	22
	Median(25%, 75%)	Median(25%, 75%)	Median(25%, 75%)	Median(25%, 75%)
Glu_0_ *	192(145, 252)	236(166, 302)	254(201, 337)	264(174, 374)
Glu_1 h_– Glu_0_	8(−8, 25)	7(−6, 31)	60(−45, 64)	−7(−34, 14)
Glu_0–1 h_ *	191(153, 261)	228(171, 310)	291(233, 359)	249(166, 361)
Glu_0–1 h_ – Glu_0_ *	2(−6, 12)	1(−7, 14)	32(22, 37)	−4(−22, 13)
(Glu_1 h_– Glu_0_)/Glu_0_(%)	5(−5, 17)	4(−3, 13)	25(−13,30 )	−3(−11, 8)
(Glu_0–1 h_ – Glu_0_)/Glu_0_(%)*	1(−3, 8)	0(−3, 7)	15(7, 16)	−2(−9, 6)

* *P*<0.05 with non-parametric *t*-test.

Glu_1 h_– Glu_0_: Glucose increment at 1 h.

Glu_0–1 h_: Mean glucose level within 1 h of fresh dialysate exchange.

Glu_0–1 h_ – Glu_0_: Mean increment of glucose levels within 1 h.

**Figure 4:**
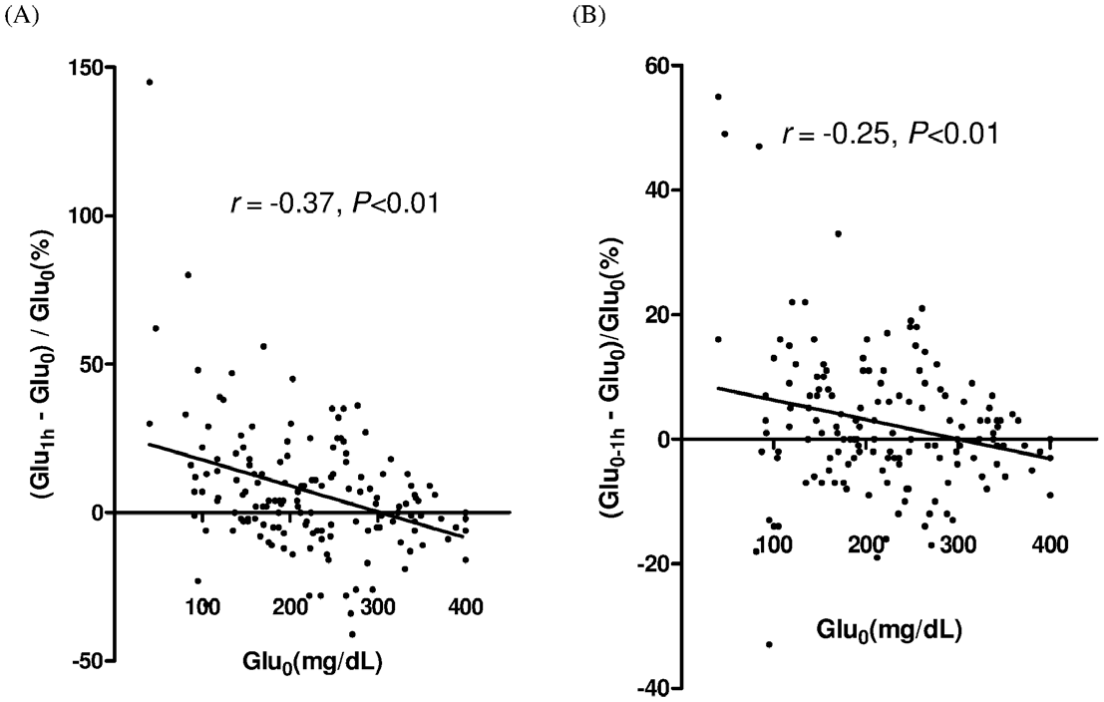
Glucemic change within the first hour of dialysate exchange. Within the first hour of peritoneal dialysis (PD) fluid exchange, correlation between Glu0 and (Glu1 h – Glu0)/Glu0 (A) and (Glu0–1 h – Glu0)/Glu0 (B).

The above results suggested that baseline Glu_0_ was higher when patients used high glucose concentration dialysate. The Pearson correlation was used to clarify this relationship, and a reverse correlation between Glu_0_ and (Glu_1 h_ – Glu_0_) (r = –0.22, *P*<0.01) or (Glu_0–1 h_ – Glu_0_) (r = –0.16, *P* = 0.05) was found. When the extent of changes in glucose concentration were further analyzed, (Glu_1 h_ – Glu_0_)/Glu_0_ and (Glu_0–1 h_ – Glu_0_)/Glu_0_ were significantly inversely correlated with baseline glucose levels (r = –0.37, *P*<0.01 and r = –0.25, *P*<0.01, respectively, Figure 4).

### Peritoneal Membrane Transport Characeristics and Glycemic Control Parameters

Since dialysate dextrose concentration was correlated with peritoneal membrane transport function, we examined the correlation between PET and glycemic control parameters. 4 hrD/P creatinine was correlated with HbA1c (r = 0.41, *P*<0.05), fructosamine (r = 0.41, *P*<0.05), AlbF (r = 0.56, *P*<0.01), and mean AUC of CGMS (r = 0.53, *P*<0.01).

We also examined PET on the changes in glucose levels within the first hour of dialysate exchange. 4 hrD/P creatinine was correlated with (Glu_0_) (r = 0.57, *P*<0.05) or (Glu_0–1 h_) (r = 0.62, *P*<0.01) and D4/D0 glucose was inversely correlated with (Glu_1 h_-Glu_0_) (r = −0.51, *P*<0.05) or (Glu_0–1 h_-Glu_0_) (r = −0.48, *P*<0.05).

### Effects of Dialysate on HbA1c

Since HbA1c was positively correlated with the mean ISF glucose when using CGMS, we divided the participants into 2 groups according to median HbA1c levels. One group had HbA1c levels <8% (n = 12), and the other group had HbA1c levels ≥8% (n = 13). We analyzed the effect of dialysates on glucose levels of the 2 groups. Mean glucose concentration within the first hour (Glu_0–1 h_) of PD fluid exchange was found to be higher in the high HbA1c group with marginal significance (*P = *0.05, Table 4). However, there was no difference in absolute value or extent of glucose increment, Glu_1 h_ – Glu_0_ or Glu_0–1 h_ – Glu_0_, between these 2 groups (Table 4).

**Table 4:** Comparison of ISF glucose concentration change within the first hour of PD fluid exchange between patients with HbA1c levels <8% and ≧8%.

	HbA1c <8(n = 12)	HbA1c ≧ 8(n = 13)	
Dialysis modality (CAPD/APD)	9/3	7/6	
The first hour after PD fluid exchange in CAPD patients			
Glu_0_ (mg/dL)	191(153, 258)	249(183, 294)	*P = *0.086
Glu_0–1 h_ (mg/dL)	198(159, 264)	273(182, 299)	*P = *0.050
Glu_1 h_– Glu_0_ (mg/dL)	11(1, 18)	15(7, 22)	*P = *0.414
Glu_0–1 h_ – Glu_0_ (mg/dL)	4(−2, 8)	6(−1, 12)	*P = *0.221
(Glu_1 h_– Glu_0_)/Glu_0_ (%)	8(1, 15)	5(3, 16)	*P = *0.806
(Glu_0–1 h_ – Glu_0_)/Glu_0_ (%)	3(−95, 70)	2(−18, 10)	*P = *0.514
Single fasting glucose	179(136, 242)	156(115, 280)	*P = *0.644
Mean ISF glucose*	188(155, 225)	236(188, 291)	*P = *0.039

* *P*<0.05 with non-parametric *t*-test.

Interstitial fluid (ISF).

## Discussion

In this study, HbA1c and AlbF levels were found to be reliable indices of glycemic control in DM patients receiving PD since these levels were significantly correlated with the mean ISF glucose levels when CGMS was used as a standard of chronic glycemic control. In addition, CGMS demonstrated that icodextrin dialysate had more favorable glycemic effect than other glucose-based dialysates.

Traditional criteria for DM diagnosis are based on spot values of blood glucose levels. However, one study found that spot values were not associated with chronic glycemic control, and there was no association of spot values with DM complications [26]. HbA1c levels, which reflect chronic blood glucose concentration, are a better index of overall glycemic control and provide risk assessment for long-term complications [27]. However, HbA1c levels can be lower along with reduced red cell survival by uremic toxin as well as increased turnover by erythropoietic agents [4]. On the other hand, they become higher under high carbonyl stress in uremia milieu [5]–[10]. Therefore, it is still not known whether HbA1C can accurately assess glycemic control in CKD patients.

Using the CGMS, we found that HbA1c, AlbF, and fructosamine were good markers of glycemic control in DM PD patients; this was especially true for HbA1c and AlbF. These 2 parameters represented the mean glucose levels better than others, and this finding was inconsistent with other studies that reported that HbA1c levels, as compared to GA%, underestimated glycemic control in dialysis patients [13], [14], [28]. However, in those studies, either random serum glucose concentration or the mean of monthly fasting serum glucose concentrations were used as the standard of comparison. In our study, on the other hand, single fasting glucose level was correlated with mean ISF glucose level with only marginal significance (Figure 2). It is, therefore, not appropriate to use single fasting sugar level as a standard to monitor glycemic control in DM PD patients.

For PD patients, protein loss from effluent about 5 to 15 g daily with little variation [29]. However, both glycated and non-glycated protein will exist in effluent with similar ratio as in blood. Therefore, AlbF and GA% will not be affected by the extent of protein loss. This result might explain that the AlbF might be more accurate than fructosamine alone to represent glycemic control. For GA, an inappropriate glycemic index in this study, molecular weight is higher than that of fructosamine (179 daltons) and the measurement methods for fructosamine and GA are quite different. We are not sure whether the result is related to the methodology we applied. A number of previous studies showed that icodextrin reduced the burden of glucose overexposure and facilitated glycemic control in DM patients [30], [31]. Our results further confirmed that glucose levels increased approximately 4–5% at first hour after exchanging conventional glucose-based dialysate. CGMS could clearly demonstrated that icodextrin dialysate had no effect or even lowered ISF glucose levels. This result reinforced the advantages of icodextrin-based dialysate use in DM patients.

The reverse relationship between Glu_0_ and glucose changes (Glu_1 h_ – Glu_0_)/Glu_0_ and (Glu_0–1 h_ – Glu_0_)/Glu_0_ indicated that higher baseline glucose levels reflected smaller changes of ISF glucose levels after PD fluid exchange (Figure 4). These results could explain the similar magnitude of increment in glucemic levels for 1.36% and 2.25% glucose dialysate since the Glu_0_ levels in the 2.25% dialysate were higher than those of the 1.36% dialysate (Table 3).

Poor sugar control forced diabetic PD patients to higher glucose dialysate to maintain ultrafiltration with subsequent peritoneum damage and higher peritoneal transporting capacity. Therefore, 4 hrD/P creatinine was correlated with HbA1c, fructosamine, AlbF, and mean AUC of CGMS. The lower D4/D0 glucose indicated the higher glucose absorption and was inversely correlated with the increment of glucose levels after refilling dialysate.

Finally, for the patients with HbA1c <8% and ≥8%, Glu_0–1 h_ appeared to be higher in the HbA1c ≥8% group with marginal significance. However, there was no difference in (Glu_0–1 h_ – Glu_0_) or (Glu_0–1 h_ – Glu_0_)/Glu_0_ between these 2 groups. Baseline glucose levels (Glu_0_) might play a major role in Glu_0–1 h_ levels, and the glucose load from dialysate had a fixed extent of increment of glucose in each patient (Table 4). Reduced Glu_0_ may be more important in the reduction of HbA1c. Higher glucose levels will need more high glucose dialysate usage to facilitate ultratfiltration and might also contribute to elevating the Glu_0_ levels. Perhaps, the vicious cycle could be broken with icodextrin dialysate. In Taiwan, the insurance regulation restricts icodextrin dialysate usage in HbA1C more than 7% or using more than a half of high glucose (≥2.25%) dialysate use. So the baseline Glu_0_ was higher in the data collected for the icodextrin. On the other hand, use of icodextrin dilysate to achieve adequate ultrafiltration can avoid the further elevation in glucose levels by using high glucose dialysate.

There are limitations in this study. First, we enrolled 25 patients, but only 16 patients received CAPD. For those patients who received automated PD with a cycler, dialysate glucose concentration could not accurately define if the PD fluid containing mixed different glucose concentration dialysate. Second, we conducted only a cross-sectional study and lack of a prospective follow-up. Third, CGMS can only record 3-day glucose levels; it remains unclear whether these data can represent 3 weeks for fructosamine or even 3 months for HbA1 levels. However, 3-day serial measurement by CGMS is more accurate than spot blood glucose levels. Finally, there was no uniform prescription for each patient, so we can’t have the same sample numbers of different dialysates in the same patients, however, this will be an universal limitation in the PD dialysate study.

In conclusion, serial measurement of 3-day glucose levels by using CGMS demonstrated that HbA1c and AlbF are appropriate indices of glycemic control in DM patients receiving PD. Higher baseline glucose levels indicate higher glucose concentration dialysate usage with a vicious cycle. Icodextrin-based dialysate which provides a more favorable glycemic control effect.might play a role to brake the cycle.
